# Supplementary material for: Patient, parent and provider perspectives on sickle cell disease genetics research in Jamaica
Source: PLOS Glob Public Health. 2026 Jan 23;6(1):e0005789. doi: 10.1371/journal.pgph.0005789 (PMC12829944; doi:10.1371/journal.pgph.0005789)
Supplement: S1 Appendix — (DOCX) [file pgph.0005789.s001.docx]

S1 Appendix: In-depth Interview Guides

| Healthcare Provider In-depth Interview Guide | | |
| --- | --- | --- |
| Question | Focus of Question Related to Methods or study Aims | Probes |
| *Part 1- For this first set of questions, I want to get a general sense of your work with sickle cell disease* | | |
| When did you start working with patients with SCD? | To build rapport | What does a normal day working with SCD patients look like for you? |
| Where did your interest in working with patients with sickle cell disease come from? | To build rapport; to gain a sense of participant’s interests and expertise | Can you describe your academic and professional journey that led you here? Can you tell me about any personal experiences you have had with sickle cell disease? |
| Tell me about some of the patients you see most frequently | To build rapport; to ease the participant into talking about sickle cell disease experiences | Can you tell me about any cases that have stood out to you?  What are some of the main challenges your patients face? (Physically and emotionally) |
| What kind of self- management techniques are you familiar with among patients? | To get a better understanding of participant’s professional experiences with the disease | What are some techniques that you have recommended to your patients? |
| What are some of ways that your patients cope emotionally with having the disease? | To get a better understanding of participant’s professional experiences with the disease | Are there any that you find concerning?  What do you tend to recommend? |
| *Part 2- Genetics and genomics research have attracted a lot of attention in recent years. For this next set of questions, I want to talk with you about your understanding of genetics research and some of the issues that might be associated with it. These include sample collection, storage and sharing. I also would like to talk about why you think some people might take part in these kinds of research. This is all still quite new, and most people don’t know much about it. So again, it’s okay if you don’t know the answers, and if something isn’t clear, feel free to let me know and ask any questions you need to.*  *-These questions are adapted from the Sickle Cell Disease Genomics Network in Africa’s (SickleGenAfrica) community engagement format and survey* | | |
| To start, what do you think of when you hear the term genetics research? | To get an understanding of how participant’s conceptualize genetics research | Are there any personal stories that come to mind?  Are there any professional ones?  What is the purpose of genetics research? |
| *If unfamiliar: Genetics research studies the roles your genes play in certain traits, like the color of your eyes, the color of your hair development of certain diseases.* | | |
| How do feel about researchers having access to all the information stored in a participant’s genes? | To get an understanding of participant’s perspectives on genetics research | Do you have any concerns about this? |
| Why do you think a person (with sickle cell disease) might participate in this kind of research? |  | What are some reasons people would not want to participate? |
| Do you think that it’s important for Jamaicans to participate in research? |  | Who do you think should benefit first from results that come out of genetics research? |
| How would you decide about whether you would recommend for a patient to participate in genetics research? | To get an understanding of participant’s perspectives on genetics research | Are there cases where you would absolutely recommend against it? |
| In your experience, how do individuals living with sickle cell disease make decisions regarding the management or treatment of their illness? | To investigate ideas of autonomy, informed consent and shared decision making | Who do you find has the most influence in this respect? |
| *Hospitals and Researchers collect samples like blood, hair, saliva, urine for genetics research.* | | |
| What kind of samples do you think a person with sickle cell disease would feel most comfortable providing? | To investigate perspectives on sample collection, management and storage | Which ones would you think of as off limits?  How do you think the kind of sample a study collects influences a person’s decision to participate in genetics research? |
| *Researchers may also store samples for many years in biobanks. In the future, different researchers or organizations like pharmaceutical companies, insurance companies or even governments, may be able to go to these biobanks and take out these samples and use them in their own research or in other ways* | | |
| What do you think about storing samples in biobanks? |  | Do you have any concerns about this?  What rights should participants have over these samples?  Control, benefits? |
| What do you think about storing samples from Jamaican participants in biobanks located overseas? |  | How do you think this could impact the Jamaican participant’s rights? |
| Should there be a limit for how long samples can be stored? | To investigate perspectives on sample collection, manage and storage | What do you think about samples being stored indefinitely? |
| How do you feel about researchers or other organizations in Jamaica or other countries being able to get and use samples stored in a biobank? | To investigate perspectives on sample collection, manage and storage | Is there any group or organization that you feel should not be able to access participant’s sample? |
| How do you feel about researchers using these samples in many studies or in many different ways? | To investigate perspectives on sample collection, manage and storage | Are there things you wouldn’t want these samples to be used for? |
| *Part 3- For this third set of questions, I would like us to discuss how you understand and what you think about existing treatments and the potential cures for sickle cell disease.* | | |
| What does good health mean to you? | To investigate the ways in which participants think about health | Do you think anyone can achieve good health?  What are some of the ways you recommend that someone with SCD try to achieve good health? |
| There is a great deal of variation in symptoms and illness severity in people with sickle cell disease. What do you think accounts for this? | To investigate the ways in which participants think about health | Do you think that social or interpersonal factors can play a role in this? |
| Can you tell me about the current treatment options available for sickle cell disease? | To assess participants understanding of and perspectives on treatments for sickle cell disease | Can you tell me about the main side effects of these treatments?  What have your patients told you about their ability to keep up with these treatments? |
| How would you define a cure for sickle cell disease? | To investigate participants’ understanding of and interest in a cure for sickle cell disease | What impact do you think it could have on a patient’s life?  What challenges do you think would remain?  Do you think there would be any new challenges that could arise? |
| *Currently, bone marrow transplantation is the only approved cure for sickle cell disease.* | | |
| Do you know of any patients that have undergone bone marrow transplantation? | To investigate participant’s experiences with sickle cell disease | If yes, what was the patient’s experience like?  If no, have you heard anything about it or come across it in any other way? |
| *There are 2 cures for SCD that are currently being developed called gene editing and gene transfer therapy that change your genes or how they are expressed.* | | |
| What do you think about treatments that make changes to a person’s genes? |  | What are some concerns you may have? |
| Would you recommend these treatments to your patients while they are still in clinical trials? | To investigate participants understanding of and interest in a cure for sickle cell disease | What are some things you think a patient would need to know about these kinds of treatments? |
| To your knowledge, is there any infrastructure in place that would allow Jamaican patients to benefit from these treatments? | To investigate participants understanding of and interest in a cure for sickle cell disease | Do you think Jamaican patients are likely to benefit from these cures?  In your opinion, what is the main factor that would determine whether Jamaican patients would benefit from these cures?  Do you think these cures would make much difference in the Jamaican setting? Why or Why not? |
| *Part 4- This final set of questions go into how people with sickle cell disease are treated in your community.* | | |
| In your experience, are people with sickle cell disease treated differently from other people in Jamaica | To explore the treatment of individuals with sickle cell disease | Can you tell me of any instances in which you witnessed a person with sickle cell disease being treated differently or unfairly because of the disease?  Can you tell me of any instances in which you witnessed a parent being treated differently?  What are some of the things that people say about sickle cell disease? |
| What if any systems are put in place to protect people living with sickle cell disease from discrimination or other kinds mistreatment? | To further explore the treatment of individuals with sickle cell disease | Do you think these systems in Jamaica are sufficient? |
| Would you say that most of your patients with SCD come in with an adequate understanding of the disease? | To explore knowledge of SCD among patients | In your experience, how do people typically learn about sickle cell disease?  What misconceptions do you most often encounter? |
| What, if anything, have you told your patients about disclosure of their sickle cell disease status? | To explore the treatment of individuals with sickle cell disease | What role do you think a support system plays in SCD management?  What, in your experience, is the largest source of support for individuals living with SCD? |
| *Finally, do you have any questions or anything else you’d like to add?*  *Thank you for taking the time to speak with me today* | | |

| Patient In-Depth Interview Guide | | |
| --- | --- | --- |
| Question | Focus of Question Related to Methods or study Aims | Probes |
| *Part 1- First, I want to get a general sense of your experiences with sickle cell disease, and here at the sickle cell unit. Just answer these questions as fully as you can and if you’re not sure about a question, please don’t hesitate to ask for clarification.* | | |
| When did you first learn you had sickle cell disease? | To get a better understanding of participant’s personal experiences with the disease | How did you learn about it?  How was your sickle cell disease explained to you for the first time?  How does a person get sickle cell disease?  Are there any interesting things you’ve heard about how a person gets sickle cell disease?  Do you remember how you felt in that moment? Did you feel supported? |
| Please tell me about some of your symptoms | To get a better understanding of participant’s personal experiences with the disease | Can you tell me how you’ve managed these symptoms?  How did you come to do these things?  What’s worked?  What hasn’t? |
| Tell me about when you first started attending the sickle cell unit. | To build rapport; to ease the participant into talking about sickle cell disease experiences | How often do you come in to the sickle cell unit?  What are some of the services you receive here? |
| Where do you turn to for information about sickle cell disease? |  |  |
| *Part 2- For this next set of questions, I want to talk with you about your understanding of genetics research and some of the issues that might be associated with it. These include sample collection, storage and sharing. I also would like to talk about why you think some people might take part in these kinds of research. This is all still quite new, and most people don’t know much about it. So again, it’s okay if you don’t know the answers, and if something isn’t clear, feel free to let me know and ask any questions you need to.*  *-These questions are adapted from the Sickle Cell Disease Genomics Network in Africa’s (SickleGenAfrica) community engagement format and survey* | | |
| To start, what do you think of when you hear the term genetics research? | To get an understanding of how participant’s conceptualize genetics research | Are there any personal stories that come to mind? |
| If unfamiliar: Genetics research studies the roles your genes play in certain traits, like the color of your eyes, the color of your hair development of certain diseases. Blood relatives often share genes, which explains why parents and their children may look alike or why family members may have certain diseases in common. | | |
| Your genes store information about your body. How do you feel about researchers having access to that information? | To investigate perspectives on sample collection, management and storage | Would you want researchers to contact you to tell you about other things they might find in your genes?  Why (not)? |
| Why do you think a person (with sickle cell disease) might participate in this kind of research? | To get an understanding of participant’s perspectives on genetics research | What are some of the things that could keep people from participating in this kind of research? |
| What are some reasons that you would participate in genetics research? | To investigate ideas of autonomy, informed consent and shared decision making | Is there anyone who you normally turn to for advice about how to manage your illness?  Would they influence your decision to participate in genetics research?  What are some concerns you would have about participating in genetics research? |
| Sometimes, the results of genetics research may not be available for a while. Would you want to participate in this research if the results would not be available for many years? | To investigate motivations perspectives on participation in genetics research | Who do you think should benefit from research you participate in? |
| *Hospitals and Researchers collect samples like blood, hair, saliva, urine for genetics research.* | | |
| If you were to participate in this kind of research, what kinds of samples would you feel most comfortable providing? | To investigate perspectives on sample collection, management and storage | Why?  Which ones would you think of as off limits?  Would the kind of sample a study collects influence your decision to participate in genetics research? |
| *Researchers may also store samples for many years in places called biobanks. A biobank is a bank where, instead of money, it stores people’s biological samples. In the future, different researchers or organizations like pharmaceutical companies that make medicines, insurance companies or even governments, may be able to go to these biobanks and take out these samples (like taking out money at a bank) and use them in their own research or in other ways* | | |
| How do you feel about your samples being stored in a biobank? | To investigate perspectives on sample collection, management and storage | Are there certain rights or powers you would want to have over these samples?  Control? Benefits? |
| Would you want there to be a limit for how long your samples are stored? | To investigate perspectives on sample collection, management and storage | Why did you suggest that amount of time?  What should be done with them after this time?  Should researchers inform participants when they destroy these samples? |
| These biobanks may be overseas, like in the US or the UK. How do you feel about your samples being stored in a different country? | To investigate perspectives on sample collection, management and storage | How do you think this could affect you?  Do you think it would affect your rights as a donor?  Do you think it would affect your ability to get any benefits that may come from the research?  Do you think it would affect you being able to have a say in what happened with the samples? |
| How do you feel about researchers or other organizations in Jamaica or other countries being able to get and use samples stored in a biobank? | To investigate perspectives on sample collection, management and storage | Is there any person or group who you would not want to be able to use your samples? |
| How do you feel about researchers using these samples in many studies or in many different ways? | To investigate perspectives on sample collection, management and storage | Are there things you wouldn’t want these samples to be used for? |
| *Part 3- For this third set of questions, I would like us to discuss how you understand and what you think about existing treatments and the potential cures for sickle cell disease.* | | |
| What does good health mean to you? | To investigate the ways in which participants think about health | Do you think anyone can achieve good health?  What are some of the ways you try to have good health? |
| What are some of the treatment options for sickle cell disease that you know of? | To assess participants understanding of treatments for sickle cell disease | How many of these have you tried?  Tell me about some of the ones you’ve preferred  Tell me about some of the ones you didn’t try or prefer |
| Can you tell me about some of your experiences with the treatments you currently receive? | To investigate participants experiences with the available treatment options. | How do you keep up with these treatments?  What are some of the challenges you face? |
| How would you describe a cure for sickle cell disease? | To investigate participants understanding of and interest in a cure for sickle cell disease | What would it do?  What impact do you think it would have on your life?  Would you be interested in having access to a cure?  Why (Not)? |
| *Currently, bone marrow transplantation is the only approved cure for sickle cell disease. Bone marrow makes blood in the body. A donor gives his or her healthy bone marrow to the patient so that he or she can make healthy blood cells.* | | |
| Have you heard about bone marrow transplantation? | To investigate understanding of and perspectives on existing cures for SCD | Is it something that you want to do?  Do you think that it’s something that you could have access to?  Why? |
| *There are 2 cures for SCD that are currently being developed called gene editing and gene transfer therapy. They make changes to your genes that allow your body to make blood cells that don’t sickle. They are still being tested in research studies to see how they how they work on people with sickle cell disease.* | | |
| What do you think about treatments that could change your genes? | To investigate participants understanding of and interest in a cure for sickle cell disease | What are some concerns you may have?  What are some things you would want to know about these kinds of treatments? |
| Would you want to be a part of the research that tests these cures? | To investigate participants understanding of and interest in a cure for sickle cell disease | Why or why not? |
| Would you want to have these kinds of treatments? | To investigate participants understanding of and interest in a cure for sickle cell disease | Why or why not?  Do you think that there are resources in Jamaica for people to get these cures? |
| *Part 4- This final set of questions go into how people with sickle cell disease are treated in your community.* | | |
| Do you feel you are treated differently as someone with sickle cell disease? | To explore the treatment of parents of individuals with sickle cell disease | In what ways? Positive or negative?  What are some of the things people say?  How do you feel about this?  Do you believe any of them are true? |
| Tell me about how you’ve decided to tell people about your sickle cell disease status? | To explore the treatment of individuals with sickle cell disease | Are there any times when you’ve done this that have stood out to you?  How did they react?  Afterwards, how did you feel about your decision? |
| How does sickle cell disease affect different aspects of your life? | To investigate the participant’s perception of sickle cell disease in relation to social and cultural contexts | Does it limit you in any way?  Do you think that there are any opportunities that SCD has caused you to miss out on?  How have you tried to overcome these limits? |
| Do you have a support system (family, friends, religious groups)? |  | If yes, what role do they play in your coping? |
| How do you imagine your future? | Broad question to investigate the participant’s outlook with sickle cell disease; coping |  |
| *Finally, do you have any questions or anything else you’d like to add?*  *Thank you again for taking the time to talk with me today* | | |

| **Parent In-depth Interview Guide** | | |
| --- | --- | --- |
| **Question** | **Focus of Question Related to Methods or study Aims** | **Probes** |
| ***Part 1- First, I want to get a general sense of your and your child’s experiences with sickle cell disease, and here at the sickle cell unit. Just answer these questions as fully as you can and if you’re not sure about a question, please don’t hesitate to ask for clarification.*** | | |
| What year was your child born? | To build rapport; to ease the participant into talking about sickle cell disease experiences |  |
| Tell me about when you first learned your child had sickle cell disease? | To build rapport; to ease the participant into talking about sickle cell disease experiences | How did you feel about how the information was presented to you? Was it clear?  How does a person get sickle cell disease?  Did you feel supported in that moment? |
| Have you told your child that he or she has sickle cell disease? | To get a better understanding of participant’s personal experiences with the disease | If no, why not?  Have you decided when you will tell your child?  If yes, can you tell me about how you made the decision.  Do you remember how you felt in that moment?  How did your child react?  Did he/she understand? |
| Please tell me about some of your child’s symptoms | To get a better understanding of participant’s personal experiences with the disease | Can you tell me how you’ve managed these symptoms?  How did you come to do these things?  What’s worked?  What hasn’t? |
| When did you first begin to take your child to the sickle cell clinic? | To build rapport; to ease the participant into talking about sickle cell disease experiences | How often do you come into to the sickle cell clinic?  What are some of the services you receive here? |
| ***Part 2- For this next set of questions, I want to talk with you about your understanding of genetics research and some of the issues that might be associated with it. These include sample collection, storage and sharing. I also would like to talk about why you think some people might take part in these kinds of research. This is all still quite new, and most people don’t know much about it. So again, it’s okay if you don’t know the answers, and if something isn’t clear, feel free to let me know and ask any questions you need to.***  ***-These questions are adapted from the Sickle Cell Disease Genomics Network in Africa’s (SickleGenAfrica) community engagement format and survey*** | | |
| To start, what do you think of when you hear the term genetics research? | To get an understanding of how participant’s conceptualize genetics research | Are there any personal stories that come to mind? |
| If unfamiliar: Genetics research studies the roles your genes play in certain traits, like the color of your eyes, the color of your hair development of certain diseases. Blood relatives often share genes, which explains why parents and their children may look alike or why family members may have certain diseases in common. | | |
| Your genes store information about your body, your relatives or your heritage/ where you come from. How do you feel about researchers having access to this information? |  | Would you want researchers to contact you to tell you about other things they might find in your child’s genes? |
| Why do you think a person (with sickle cell disease) might participate in this kind of research? | To get an understanding of participant’s perspectives on genetics research | What are some of the things that could keep people from participating in this kind of research? |
| What are some reasons that would cause you to allow your child to participate in genetics research? | To investigate ideas of autonomy, informed consent and shared decision making | Is there anyone that you usually go to for advice on taking care of your child?  Would they influence your decision to participate in genetics research?  What are some concerns you would have about your child participating in genetics research? |
| Sometimes, the results of genetics research may not be available for a while. Would you allow your child to participate in this research if the results would not be available for many years? | To investigate motivations perspectives on participation in genetics research | Who do you think should benefit from research your child participates in? |
| *Hospitals and Researchers collect samples like blood, hair, saliva, urine for genetics research.* | | |
| If your child were to participate in this kind of research, what kinds of samples would you feel most comfortable with your child providing? | To investigate perspectives on sample collection, management and storage | Why?  Which ones would you think of as off limits?  Would the kind of sample a study collects influence your decision to allow your child to participate in genetics research? |
| *Researchers may also store samples for many years in places called biobanks. A biobank is a bank where, instead of money, it stores people’s biological samples. In the future, different researchers or organizations like pharmaceutical companies that make medicines, insurance companies or even governments, may be able to go to these biobanks and take out these samples (like taking out money at a bank) and use them in their own research or in other ways* | | |
| How do you feel about your child’s samples being stored in a biobank? | To investigate perspectives on sample collection, management and storage | Are there certain rights or powers you would want to have over these samples?  Control? Benefits? |
| Would you want there to be a time limit for how long your child’s sample can stay in the biobank? |  | What should be done with them after this time? |
| These biobanks may be overseas, like in the US or the UK. How do you feel about your child’s samples being stored in a different country? | To investigate perspectives on sample collection, management and storage | How do you think this could affect you or your child?  Do you think it would affect your or child’s rights as a donor?  Do you think it would affect your ability to get any benefits that may come from the research?  Do you think it would affect you being able to have a say in what happened with the samples? |
| How do you feel about researchers or other organizations in Jamaica or other countries being able to get and use samples stored in a biobank? | To investigate perspectives on sample collection, management and storage | Is there any group that you would not want to be able to get or use your child’s sample? |
| How do you feel about researchers using these samples in many studies or in many different ways? | To investigate perspectives on sample collection, management and storage | Are there things you wouldn’t want these samples to be used for? |
| ***Part 3- For this third set of questions, I would like us to discuss how you understand and what you think about existing treatments and the potential cures for sickle cell disease.*** | | |
| What does good health mean to you? | To investigate the ways in which participants think about health | Do you think anyone can achieve good health?  What are some of the ways you try to have good health?  What are some of the ways you try to ensure your child has good health? |
| What are some of the treatment options for sickle cell disease you know about? | To assess participants understanding of treatments for sickle cell disease | How many of these has your child tried?  Tell me about some of the ones you’ve preferred  Tell me about some of the ones you didn’t try or prefer |
| Can you tell me about some of your experiences with the treatments your child currently receives? | To investigate participants experiences with the available treatment options. | How do you keep up with these treatments?  What are some of the challenges you face? |
| How would you describe a cure for sickle cell disease? | To investigate participants understanding of and interest in a cure for sickle cell disease | What would it do?  What impact do you think it would have on your child’s life? |
| *Currently, bone marrow transplantation is the only approved cure for sickle cell disease. Bone marrow makes blood in the body. A donor gives his or her healthy bone marrow to the patient so that he or she can make healthy blood cells.* | | |
| Have you heard about bone marrow transplantation? |  | Is it something that you want for your child?  Do you think that it’s something that you could have access to?  Why? |
| *There are 2 cures for SCD that are currently being developed called gene editing and gene transfer therapy. They make changes to your genes that allow your body to make blood cells that don’t sickle. They are still being tested in research studies to see how they work on people with sickle cell disease* | | |
| What do you think about treatments that could change your child’s genes? | To investigate participants understanding of and interest in a cure for sickle cell disease | What are some concerns you may have?  What are some things you would want to know about these kinds of treatments? |
| Would you let your child have these kinds of treatment? | To investigate participants understanding of and interest in a cure for sickle cell disease | Why or why not? |
| ***Part 4- For these final questions I want to go into how people with sickle cell disease are treated in your community.*** | | |
| Do you feel you are treated differently as the parent of a child with sickle cell disease? | To explore the treatment of parents of individuals with sickle cell disease | In what ways? Positive or negative?  What are some of the things people say?  How does this make you feel?  Do you feel that any of them are true? |
| What do you tell your child about telling others about his or her sickle cell disease status? | To explore the treatment of individuals with sickle cell disease | How do you decide to tell others about your child’s sickle cell disease status?  Can you tell me about any moments relating to this that have stood out to you? |
| How do you think sickle cell disease may affect his or her life? | To investigate the participant’s perception of sickle cell disease in relation to social and cultural contexts; coping | Do you think it may limit your child in any way?  How?  Do you think that there are opportunities that sickle cell disease has caused your child to miss out on?  How have you tried to help your child overcome these limits? How do you help him or her cope? |
| How do you cope with the ways that the disease has affected your child? |  | Do you have a support system (family, friends, religious groups)?  If yes, what role do they play in your coping? |
| How do you imagine your child’s future? | Broad question to investigate the participant’s outlook with sickle cell disease |  |
| *Finally, do you have any questions or anything else you’d like to add?*  *Thank you again for taking the time to talk with me today* | | |
