# Supplementary material for: Patient, parent and provider perspectives on sickle cell disease genetics research in Jamaica
Source: PLOS Glob Public Health. 2026 Jan 23;6(1):e0005789. doi: 10.1371/journal.pgph.0005789 (PMC12829944; doi:10.1371/journal.pgph.0005789)
Supplement: S2 Appendix — (DOCX) [file pgph.0005789.s002.docx]

S2 Appendix: Code Book

| **Name** | **Description** | **Files** | **References** |
| --- | --- | --- | --- |
| Deterrents | Specific reasons participants give directly when asked about why they or others would not participate in genetics research, or reasons given in other contexts about why one wouldn’t participate in research | 27 | 91 |
| Fear | Reasons given for not wanting to participate in genetics research that include references to fear, including fears of the unknown, a fear of knowing, a fear of the research process or of pain or discomfort | 25 | 70 |
| Invasiveness | Participants express their concerns about the invasiveness or the paint that may be associated with the research process | 19 | 42 |
| Mistrust | Reasons given for not wanting to participate in genetics research that include comments that convey a mistrust of the research process or of researchers or concerns about the misuse of samples | 14 | 26 |
| Risky Trials | Concerns expressed about the risks involved with participating in trials for gene-based cures | 8 | 12 |
| The Unknown | Fear of the unknown, fear of knowing, fear of the newness of this type of research and these new techniques | 7 | 8 |
| Life Stage |  | 3 | 3 |
| Mores | Concerns influenced by certain beliefs or mores. | 2 | 2 |
| Time & Inconvenience | Reasons given for not wanting to participate in genetics research that include references to concerns about time commitment, a lack of availability. | 8 | 14 |
| Ethical | Top level code to include all ethical issues | 29 | 217 |
| Autonomy | Covers issues related autonomy in the research process including control of samples, ownership of samples, having a say in sample use and issues related to consent in general | 28 | 63 |
| A Say |  | 12 | 23 |
| Blanket |  | 11 | 12 |
| Broad |  | 2 | 2 |
| Depends |  | 2 | 3 |
| Dynamic |  | 11 | 14 |
| No Say |  | 14 | 22 |
| Specific |  | 3 | 3 |
| Collaboration | Covers issues related to protecting the participant from harm | 2 | 3 |
| Confidentiality | Issues related to ensuring participant information is kept confidential | 10 | 16 |
| Limits | Discussion surrounding how far participants belief researchers should go in the research process. What is off limits? | 15 | 30 |
| ROR | Discussions involving the return of results and benefit sharing | 23 | 67 |
| Community Benefit |  | 8 | 13 |
| No Benefit |  | 6 | 8 |
| Participant Benefit |  | 11 | 24 |
| Transparency | Discussions involving the extent to which participants want to know what happens to their samples and how they are used in research | 18 | 38 |
| Broad |  | 0 | 0 |
| None |  | 1 | 1 |
| Specific |  | 13 | 26 |
| Genetics Research Attitudes | Participant attitude towards genetics research and research in general. This would include descriptions of willingness to participate or comments about the importance of research in general, genetics research, or research involving Jamaican participants | 21 | 60 |
| Excitement and Optimism | Participants discuss genetics research with excitement and optimism about where it may lead and their willingness to participate | 10 | 18 |
| Inclusive | Participants convey the importance of Jamaicans participating in genetics research | 2 | 2 |
| Necessary | Genetics research must be done | 12 | 18 |
| Skepticism or Reluctance | Participants express skepticism or reluctance towards the research process. “Gray area” | 6 | 7 |
| What is it | How participants understand genetics research | 7 | 7 |
| Motivators | Specific reasons participants give directly when asked about why they or others would participate in genetics research, or reasons given in other contexts about why one should or would participate in research | 27 | 83 |
| Help | Reasons given for participating in genetics research that include seeking help either for themselves or for others in managing the disease, whether that be in the form of better treatment in general or a cure for the disease. | 24 | 56 |
| To Create a Better Life |  | 9 | 18 |
| For Others |  | 11 | 16 |
| For Themselves | For themselves or their children with SCD | 9 | 13 |
| Learn | Reasons given for participating in genetics research that include wanting to learn more about the disease, about themselves or others. | 17 | 27 |
| Advance Medical Knowledge |  | 7 | 7 |
| Advance Personal Knowledge |  | 10 | 12 |
| Deference | Captures comments that convey a deference or submissiveness to or an attitude of practicality towards the research process. 'it is what it is' or 'as long as it helps' attitudes | 24 | 81 |
| Powerless | Participants convey a powerlessness or out of control attitude towards the research process and are fine with it. That’s the way things are | 5 | 10 |
| Practical Attitude | An ‘as long as it helps’ or ‘whatever it takes’, or ‘whatever is needed’ mindset | 25 | 60 |
| Trust |  | 5 | 6 |
