# Supplementary material for: Patient, parent and provider perspectives on sickle cell disease genetics research in Jamaica
Source: PLOS Glob Public Health. 2026 Jan 23;6(1):e0005789. doi: 10.1371/journal.pgph.0005789 (PMC12829944; doi:10.1371/journal.pgph.0005789)
Supplement: Attachment — Submitted filename: Response_to_Reviewers.docx [file pgph.0005789.s005.docx]

November 26, 2025

Rahul Gajbhiye, MBBS PhD

Academic Editor

PLOS Global Public Health

Dear Dr. Gajbhiye,

Thank you for the quick review and request for revisions of our manuscript entitled, “Stakeholder Perspectives on Sickle Cell Disease Genetics Research in Jamaica”. We have now completed the requested revisions for both reviewers which further strengthen the impact of this work. Please find a detailed point-by-point response to the reviewers concerns and corresponding highlighted changes to the manuscript in the newly uploaded materials.

Krystin Jones, PhD

Department of Epidemiology
Bloomberg School of Public Health

Johns Hopkins University, Baltimore MD

Reviewer 1

The authors in this manuscript have described the perspectives of patients, family members and healthcare providers on role of genetics pertaining to SCD. My comments are as follows:

1. The title “Stakeholder Perspectives on Sickle Cell Disease Genetics Research in Jamaica” sounds broad but may overstate the range of perspectives included. Though the manuscript includes the perspectives of arguably the most directly affected stakeholders, the title doesn’t make this clear. A more specific title could show which groups are the focus, so readers know whose perspectives shape the analysis.

**Author’s Response: We have updated our title to more specifically indicate which stakeholders we engaged in this study:**

**Patient, Parent and Provider Perspectives on Sickle Cell Disease Genetics Research in Jamaica**

2. The framework which was used for thematic analysis should be included in the study design in the methods section.

**Author’s Response: Thank you for pointing out the need for greater clarity in our analytic methods. We conducted thematic analysis based on the methods described in Saldaña’s The Coding Manual for Qualitative Researchers. We have clarified this in the text as below:**

**“Transcripts were closely read and structurally coded by a single coder (KJ) based on interview questions and the general structure of the interview guide (Appendix 2) through thematic analysis based on the methods described in Saldaña27”**

3. Methods (Page 9, line number 162): Please clarify whether coding and thematic analysis were conducted by a single author or if codes and themes were independently reviewed/validated by a second author. Additionally, specify how data triangulation was ensured.

**Author’s Response: Thank you again for pointing out the need for clearer language. We explained in the manuscript that coding and thematic analysis were conducted by a single coder (KJ). We’ve edited the language to improve clarity. The new text reads:**

**“Transcripts were closely read and structurally coded by a single coder (KJ) based on interview questions and the general structure of the interview guide (Appendix 2) through thematic analysis based on the methods described in Saldaña27. Codes and themes were reviewed by a second author (CR).”**

4. Results (Table 1): the median needs to be reported along with interquartile range instead of range of values.

**Author’s Response: Thank you for pointing this out. We’ve now added the median and interquartile ranges.**

5. Results (Pg 17, line 303-304): The statement “theme summarized in error, reference source not found!!!” requires clarification and correction.

**Author’s Response: Thank you bringing this to our attention. We have now removed this from the text.**

Reviewer 2

In the submitted manuscript the authors have tried to understand the stakeholder’s perspectives about genetic research in sickle cell disease in Jamaica using qualitative research methods. As the possibilities for genetic treatment of sickle cell disease are growing, this becomes an important area to study.

I have the following major comment

• The study’s findings have important practice implications. In the last section of the manuscript the authors do provide some suggestion but they appear more abstract. The authors may want to provide 4 to 5 concrete suggestions based on their findings and may consider presenting these suggestions in a table. For example, the authors state- “Greater communication and understanding of research goals and the implications of research participation will be essential for protecting participant autonomy throughout the research process.” How can this be achieved? What would be mechanisms on the ground to ensure this?

**Author’s Response: We have edited the manuscript to provide clearer, more concrete practical suggestions, as shown below:**

**“We present the following practice recommendations:**

**1)	Implement community-sessions prior to study recruitment in which local clinicians, community health workers or trusted local researchers communicate research goals and processes and provide opportunities for questions in an informal setting.**

**2)	Create culturally relevant tools and materials such as booklets or videos to better convey the risk, benefits, expectations, and limitations of genetics research participation, and facilitate greater transparency and improved trust.**

**3)	Integrate Local Advisory Boards in Study Governance that includes local researchers, community advocates, patient representatives, and ethics committee members to advocate on behalf of participants and provide ongoing community feedback throughout the process to reduce power imbalances and facilitate local oversight.**

**4)	Establish clear policies that dictate data use of and return of results that communicate to participants how their data will be stored, shared, and used, what results will be returned to participants, and the process by which these results will be returned to foster trust.”**

There are some minor concerns-
• Why did the authors choose in-dept interviews over focus group discussions for patients and parents? Focus group discussions often bring out more information which more than the collective of the individual opinions. The authors may want to provide an explanation.

**Author’s Response: Thank you for this question. While we agree that focus groups may have been useful to derive information and opinions through the group setting that individual interviews may not have been able to provide alone, we felt that in-depth interviews better facilitated deeper insights and connections and created space for participants to discuss topics that may have been more sensitive or personal to them. Additionally, the need to conduct such discussions through video conferencing platforms due to the pandemic would have introduced additional logistical challenges and possibly limited their effectiveness. We now address this in new text presented below:**

**“Data was collected through individual in-depth interviews to facilitate interviewee comfort and gain deeper, individual insights on personal or sensitive topics including experiences of stigma and other challenges associated with SCD.”**

• The authors state that (lines 129, 130) “patients and parents were randomly selected from a list of patient data. A purposive sampling strategy was also used to ensure that male patients and parents were included in the study” These two sentences appear contrasting. Kindly provide further explanation.

**Author’s Response: Thank you for pointing out this discrepancy. Patients were randomly selected. However, when we realized the greater number of female patients being enrolled in the study, we began to sample from a list of male participants to ensure representation of male perspectives. We clarify this is the manuscript:**

**“From September to December 2020, Providers were recruited through convenience sampling, while patients and parents were randomly selected from a list of patient data. However, given the greater number of female patients and parents being enrolled into the study, we then implemented a purposive sampling strategy to ensure that male patients and parents were included.”**

• In qualitative studies, typically, mean and median data are not presented. The authors may want to just provide ranges in Table 1.

• In healthcare research the patients are often given least importance from a power perspective. In table 1 the authors present data on providers first followed by patients and parents. I do not know if the authors had something specific in mind while arranging the data in this way but I would suggest presenting data on patients and parents first followed by those from the providers.

**Author’s Response: Thank you for pointing this out. This is a very important point and we have now rearranged Table 1 to represent this.**

• This is an extension of my first comment. Most of the responders in the study were women which is welcome. The readers may want to know if a majority of patients attending the SCD clinic were women. Also, will this overrepresentation have implications for the findings of the study? Authors may want to discuss this in the manuscript.

**Author’s Response: Thank you for highlighting this very important point. We have addressed the overrepresentation of female participants in new text shown below:**

**“Relatedly, this study featured a greater number of female respondents compared to male respondents (72.4% vs 27.6%). This may have been due to several factors including the greater number of female providers and parents, the willingness or availability of male patients to participate in the study, among others. The study’s largely female sample may have implications for the generalizability of results, particularly in the case of SCD patients.”**

• There is a typo on line 303 which the authors should correct. “in Error! Reference source not found”

**Author’s Response: Thank you bringing this to our attention. We have now removed this from the text.**
